# Modeling Healthcare Processes Using Commitments: An Empirical Evaluation

**DOI:** 10.1371/journal.pone.0141202

**Published:** 2015-11-05

**Authors:** Pankaj R. Telang, Anup K. Kalia, Munindar P. Singh

**Affiliations:** 1 Cisco Systems Inc., Research Triangle Park, Durham, North Carolina, United States of America; 2 Department of Computer Science, NC State University, Raleigh, North Carolina, United States of America; Université Toulouse 1 Capitole, FRANCE

## Abstract

The two primary objectives of this paper are: (a) to demonstrate how Comma, a business modeling methodology based on commitments, can be applied in healthcare process modeling, and (b) to evaluate the effectiveness of such an approach in producing healthcare process models. We apply the Comma approach on a breast cancer diagnosis process adapted from an HHS committee report, and presents the results of an empirical study that compares Comma with a traditional approach based on the HL7 Messaging Standard (Traditional-HL7). Our empirical study involved 47 subjects, and two phases. In the first phase, we partitioned the subjects into two approximately equal groups. We gave each group the same requirements based on a process scenario for breast cancer diagnosis. Members of one group first applied Traditional-HL7 and then Comma whereas members of the second group first applied Comma and then Traditional-HL7—each on the above-mentioned requirements. Thus, each subject produced two models, each model being a set of UML Sequence Diagrams. In the second phase, we repartitioned the subjects into two groups with approximately equal distributions from both original groups. We developed exemplar Traditional-HL7 and Comma models; we gave one repartitioned group our Traditional-HL7 model and the other repartitioned group our Comma model. We provided the same changed set of requirements to all subjects and asked them to modify the provided exemplar model to satisfy the new requirements. We assessed solutions produced by subjects in both phases with respect to measures of flexibility, time, difficulty, objective quality, and subjective quality. Our study found that Comma is superior to Traditional-HL7 in flexibility and objective quality as validated via Student’s t-test to the 10% level of significance. Comma is a promising new approach for modeling healthcare processes. Further gains could be made through improved tooling and enhanced training of modeling personnel.

## Introduction

Modern healthcare is characterized by a network of participants such as primary care physicians, specialists, hospitals, laboratories, and insurance providers. These participants interact in complex ways to provide patient care. It has been argued that incorrect processes can lead to inefficiencies and sometimes to critical medical errors adversely impacting patient safety [1]. Therefore, it is important to develop healthcare process models that help make the work repeatable, provide a standard for monitoring and compliance, and guide the participants regarding their mutual expectations.

Healthcare processes naturally involve the participation of multiple individuals and organizations. Therefore, it is important that process models support the autonomy of the participants and the heterogeneity of their information systems. Effective process modeling is a key motivation underlying approaches such as Health Level Seven (HL7) [2], which is a leading standard for modeling healthcare processes. HL7 has contributed significantly to standardizing healthcare communications. A bulk of HL7’s contributions have been in information modeling and message schemas. HL7 Version 3 employs a Reference Information Model (RIM) [3] to promote consistency in information models across HL7 messages from different domains. Other work in healthcare Information Technology (IT) has also concentrated on information modeling from a semantic standpoint, e.g., Unified Medical Language System (UMLS) [4]. However, existing work focuses only on operational details such as messages that the participants exchange and their temporal ordering in process (interaction) models. For example, a traditional process model may specify the following temporally ordered messages: (a) a physician sends an imaging order message to a radiologist; (b) the radiologist responds with an imaging results message; and (c) the physician sends a payment message to the radiologist. In particular, operational models tend to hide the key business relationships [5], making it difficult to understand why participants exchange certain messages and why they are ordered in a certain way. For example, why does the radiologist provide imaging results upon a physician’s request? Is there a commitment from the radiologist to send the results to the physician upon request? In case of a traditional model, the modelers can assure themselves of the correctness of only a few of the possible enactments, which they codify explicitly in their models, for example, as seen in Singureanu’s ([6], pp. 45–47) examples. Hardcoding a small set of enactments limits the flexibility of the participants, precluding effective handling of unexpected situations.

We advocate that the processes be modeled in terms of business relationships rather than low-level operational details. Business relationships capture the essence of the dependencies among the participants while leaving each participant free to develop and modify its internal implementations as it sees fit. As an outcome of our formal studies into business modeling, we recently developed Comma, a methodology that models a process in terms of how the process leads to the creation and progression of interrelated commitments (explained below) among its participants [7]. Importantly, Comma is backwards compatible with traditional approaches in that it outputs the same operational representation, namely, Unified Modeling Language 2.0 Sequence Diagrams (SDs) [8, 9].

Comma has previously been applied in the commerce and manufacturing domains [7, 10]. We posit that Comma would prove effective in healthcare process modeling as well. Specifically, we claim that Comma yields flexible process models that are easier to comprehend and modify than the models that a traditional method based on HL7 (Traditional-HL7) yields. We verify our claims via an empirical study of developers that compares Comma and Traditional-HL7 on criteria including objective and subjective quality, and flexibility of the models produced.

The rest of this paper is organized as follows. The Background section introduces some relevant concepts. The Methods section applies both Comma and Traditional-HL7 to a breast cancer care scenario, seeking to demonstrate the potential benefits of high-level modeling as supported by Comma. Further, the Methods section describes our empirical study to evaluate whether the benefits alluded to above would hold when other modelers applying Comma and Traditional-HL7. The Results section presents our findings from this study. The Related Literature section reviews the relevant literature. The Discussion section concludes the paper with commentary on its results and positioning.

## Background

This section describes essential background on commitments, the Comma methodology including business patterns, and the Traditional-HL7 methodology.

### Commitments

A commitment [11–13] helps specify the relationship among business participants in a natural manner. We write a commitment from debtor to creditor to bring about the consequent if the antecedent holds as:

C(DEBTOR, CREDITOR,antecedent,consequent).

A commitment in this sense is a relational concept: it captures how its debtor and creditor *relate* to one another. It is a social (public) notion, and it does not refer to the internal beliefs or goals of any individual party. For example, the commitment from physician to patient to providing a treatment, if patient’s insurance company approves the treatment can be written as:

C(PHYSICIAN, PATIENT,insurance-approval,treatment-provided).

Note that we use small caps to indicate role names, which we treat the same as proper names.

**Fig 1:**
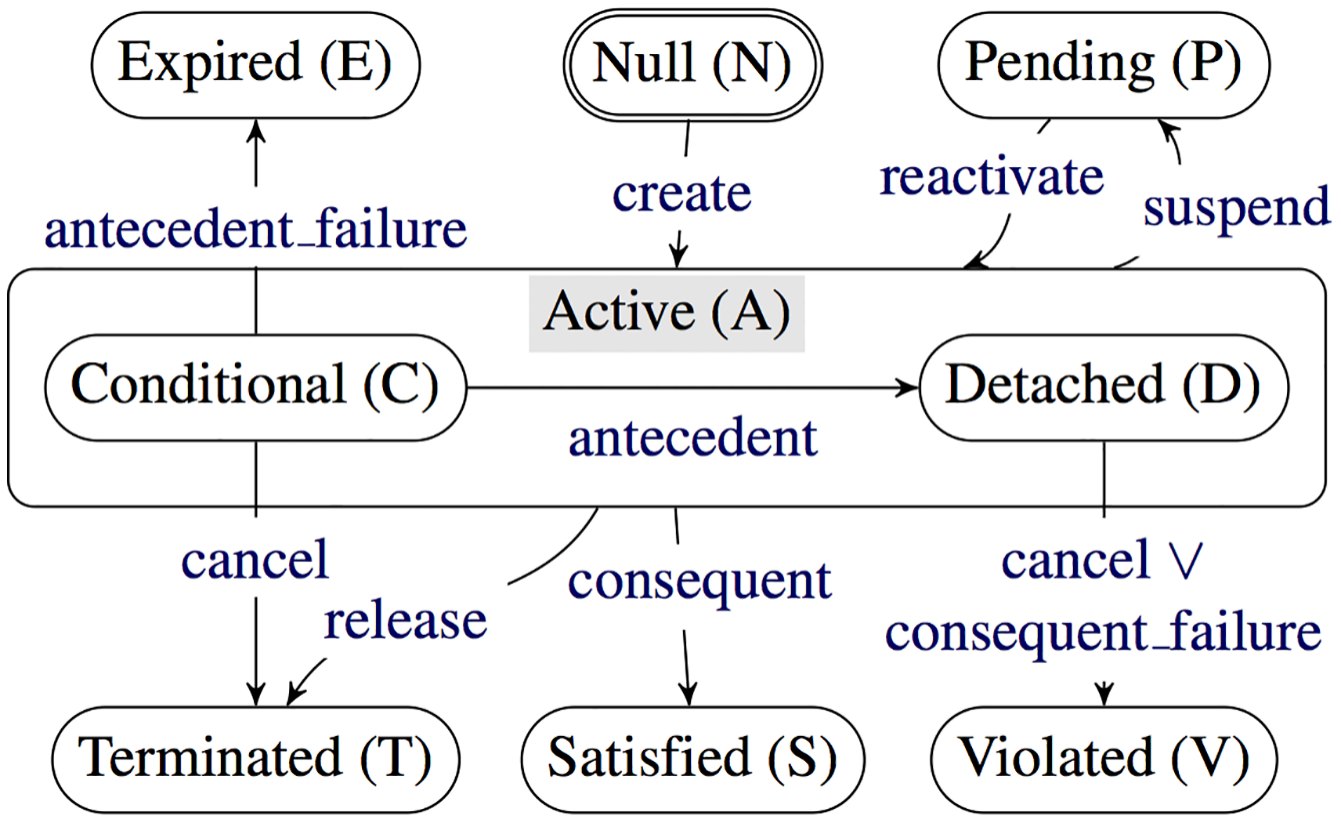
Commitment life cycle as a state transition diagram.

Fig 1 shows the lifecycle of a commitment, expressed as a UML 2.0 State Diagram [14], which we explain using the physician–patient commitment example. Upon creation by physician, the commitment transitions from null to active. The active state has two substates: conditional if the insurance company has yet to approve the treatment (that is, the antecedent does not hold), and detached if the insurance company has approved the treatment (that is, the antecedent holds). The commitment terminates if physician cancels the commitment when conditional, or patient releases physician from the commitment. The commitment is satisfied when the treatment is provided to patient (consequent holds regardless of the antecedent). The commitment is violated if the insurance company has approved the treatment (antecedent holds), and patient does not receive the treatment within a specified time bound (that is, we have consequent failure), or if physician cancels the commitment. When the commitment is conditional, if the insurance company does not approve the treatment within a specified time bound (that is, we have antecedent failure), then the commitment expires. physician may suspend the commitment, making it pending.

In general, the commitment life cycle imposes no ordering requirements. In some cases, data dependencies would impose ordering. For example, physician cannot treat patient until patient provides essential information; in other cases, there may be a legal or business prerequisite for an action. Such requirements are all compatible with the commitment itself being flexible, which helps capture the requirements modularly. Additionally, it helps to define antecedents and consequents whose truth or falsity is definite and permanent. For example, we would not ordinarily say that “the treatment is provided” but that “the treatment is provided within two business days of the insurance approval.” In other words, an appropriate timeout would be incorporated within any antecedent or consequent, e.g., treatment-provided(date(x)) where x is two days after the insurance approval date. Marengo et al. [15] describe additional subtle features of dealing with temporal constraints placed within commitments.

In existing representations, the physician-patient commitment from our example would be hardcoded into one or more message orderings. For example, physician may provide treatment after the insurance company’s approval arrives. As a result, if physician provides treatment before the insurance company’s approval, then physician would be out of *technical* compliance. However, in many cases such deviation may be appropriate and the deviating parties may remain in *business* compliance. For example, physician may elect to provide treatment even if the insurance approval is delayed. Here, physician’s motivations could be varied. For example, we might speculate that a particular physician playing the role of physician (1) estimates the probability of receiving an insurance company approval to be high and, with staff resources already paid for, estimates the expected utility of delaying treatment to be negative; (2) estimates the risk on the patient’s life to be high; (3) wishes to earn community recognition by treating a certain number of uninsured patients. In each case, physician’s early treatment satisfies his commitment so he would be fully in compliance. In sum, a participant’s business motivations can be arbitrary and the process model should only constrain behavior where it matters to a business relationship. Whereas existing approaches give prominence to operational details such as message ordering, commitments give prominence to business relationships.

### The Comma Business Modeling Methodology

Comma is based on an extensible set of business modeling patterns [5] such as *commercial transaction* and *outsourcing*. The Comma patterns are recipes of recurring business scenarios in terms of commitments. These patterns are analogous to the programming patterns such as as well known in object-oriented programming. A key difference is that the Comma patterns are high level and declarative. We envision a library of such patterns developed by the business subject matter experts. For example, in the outsourcing pattern, the outsourcer has a commitment toward a client to perform a certain task if the client pays: *C*_1_ = C(outsourcer, client, payOutsourcer, task). The outsourcer negotiates with a contractor to outsource the task that is the consequent of *C*_1_. As a result, the outsourcer commits to the contractor to pay if the contractor commits to performing the task: *C*_2_ = C(outsourcer, contractor, create(*C*_4_), payContractor), and the contractor commits to the outsourcer to creating a commitment to perform the task if the outsourcer pays: *C*_2_ = C(contractor, outsourcer, payContractor, create(*C*_4_)), where *C*_4_ = C(contractor, client, ⊤, task). For each business pattern, we specify one or more Unified Modeling Language 2.0 Sequence Diagrams (SDs) [8, 9] that operationalize that pattern. (The S1 Appendix presents the SDs for outsourcing pattern.)

We now describe the Comma methodology [7], as summarized in Table 1.

**Table 1:** Comma methodology steps.

**Step**	**Description**	**Input**	**Output**
1	Extract subscenarios	Business scenario	Subscenarios
2	Identify roles	Subscenario	Roles
3	Identify business tasks	Subscenario	Tasks
4	Introduce a Comma pattern for each subscenario	Comma pattern, subscenario, roles, tasks	Business model
5	Introduce UML Sequence Diagrams	Comma pattern Sequence Diagrams, subscenario, roles, tasks	Operational model

The Comma methodology takes a scenario description as input. Step 1 identifies discrete business interactions (subscenarios) from the scenario such that each interaction matches a business modeling pattern. Step 2 identifies the roles from the subscenario. The role name typically indicates the role’s business function. Step 3 identifies the business tasks performed by the roles. Step 4 assembles a business model from the business patterns of each subscenario. Step 5 creates an operational model by introducing Comma-specified sequence diagrams for each pattern.

### HL7 Messaging Standard

HL7 refers to a group of standards for healthcare geared toward improving patient care and optimizing workflows by reducing ambiguity. The HL7 Messaging Standard specifies messages and trigger events to support communication. Each message has a type, which describes its purpose. For example, the ADT (Admit Discharge Transfer) type includes several messages dealing with patient admission, discharge, and transfer.

In this paper, we focus on modeling a healthcare process starting from a set of messages defined in the HL7 standard. We employ the HL7 Version 2 messages. Please note that our focus is not to develop an *information model* for the messages, which is well addressed by HL7 (especially HL7 Version 3 and RIM). Instead, our focus is on developing a process (interaction) model. Therefore, we adopt a simple methodology, Traditional-HL7, that Table 2 shows. These steps are along the lines of [6], which is a well-known practical approach. Step 1 identifies the applicable messages from an HL7 catalog based on message “intent” as described in the catalog. Step 2 creates sequence diagrams that show the role (participant) names as lifelines and the messages they exchange. A sequence diagram captures the temporal ordering, constraints, and repetitions for the messages.

**Table 2:** Traditional-HL7 methodology steps.

**Step**	**Description**	**Input**	**Output**
1	Identify applicable HL7 messages based on the message intent	Business scenario	HL7 Messages
2	Develop UML Sequence Diagrams	HL7 Messages	MUL Sequence Diagrams
	1. Show the scenario participants as lifelines, and the messages they exchange		
	2. Capture the temporal ordering, constraints, and message repetitions as per the scenario requirements		
	3. Create modular sequence diagrams		

## Methods

This section applies the Comma and Traditional-HL7 methods to a breast cancer diagnosis scenario, and presents the design and results of our empirical study.

### Part One: Comparing Traditional-HL7 and Comma

We apply both approaches to the following scenario adapted from an expert committee report [16] produced by the Office of the Assistant Secretary for Planning and Evaluation (ASPE), Department of Health and Human Services. (For convenience, we use feminine pronouns for patient and radiologist and masculine pronouns for physician and pathologist below.)

patient suspects breast cancer and reports it to physician. physician collects patient’s history and examines patient for lumps or suspicious areas. physician then sends patient to radiologist for imaging. radiologist performs imaging and reports the results to physician. If physician finds patient’s condition not worrisome, physician asks patient to visit for a routine annual examination. If physician finds that patient has a benign tumor, physician asks patient to come back after four to six months. If physician finds the tumor suspicious, physician orders a biopsy. radiologist collects a tissue specimen and sends it to pathologist. pathologist examines the specimen in a laboratory and comes up with a report. pathologist and radiologist then hold a conference to ensure their results are concordant. radiologist forwards pathologist and radiologist’s integrated report to physician. pathologist communicates patient’s data to registrar, and registrar adds patient to a breast cancer registry. physician checks the integrated report and discusses the treatment steps with patient.

**Fig 2:**
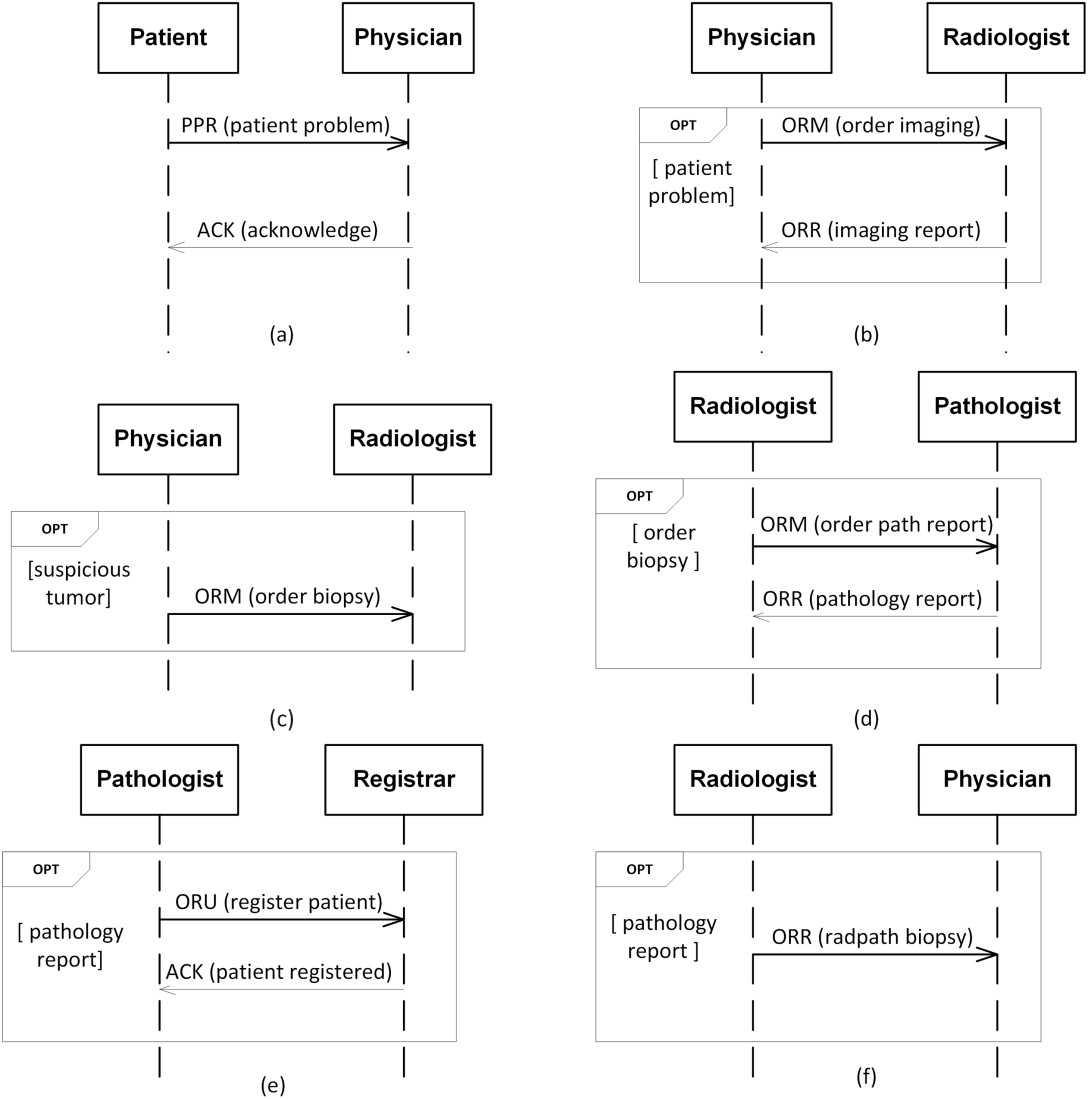
A Traditional-HL7 solution for the ASPE scenario. (a) patient requests physician to perform diagnosis. (b) physician requests radiologist to perform imaging. (c) physician requests radiologist to perform biopsy. (d) radiologist requests pathologist to provide pathology report. (e) pathologist registers patient with registrar. (f) radiologist sends radiology-pathology integrated report to physician.

Table 3 and Fig 2 respectively show the messages and sequence diagrams of a Traditional-HL7 solution to the breast cancer scenario. In Fig 2(a), patient requests physician for a checkup by sending a *patient problem* (PPR) message, and physician sends an *acknowledge* (ACK) message back to patient. In Fig 2(b), after receiving the *patient problem*, physician requests radiologist for imaging by sending a *general order* (ORM) message. radiologist delivers the imaging report by sending a *general order* response (ORR) message. In Fig 2(c), based on the *imaging report*, if physician finds the tumor suspicious, physician asks radiologist to perform a biopsy, via an *order biopsy* (ORM) message. In Fig 2(d), after sending *order biopsy*, radiologist requests pathologist to examine patient’s tissue specimen via *order path report* (ORM) message. pathologist submits his report via *pathology report* (ORR) message. In Fig 2(e), after sending *pathology report*, pathologist informs registrar about the patient via *register patient* (ORU) message, and upon registering, registrar sends *patient registered* (ACK) message to pathologist. Finally, in Fig 2(f), radiologist provides physician with patient’s biopsy report via *radpath biopsy* (ORR) message.

**Table 3:** HL7 messages applicable to the breast cancer diagnosis scenario.

**Message Name**	**Description**
PPR	Patient problem
ACK	Acknowledgment
ORM	General order request
ORR	General order response

**Fig 3:**
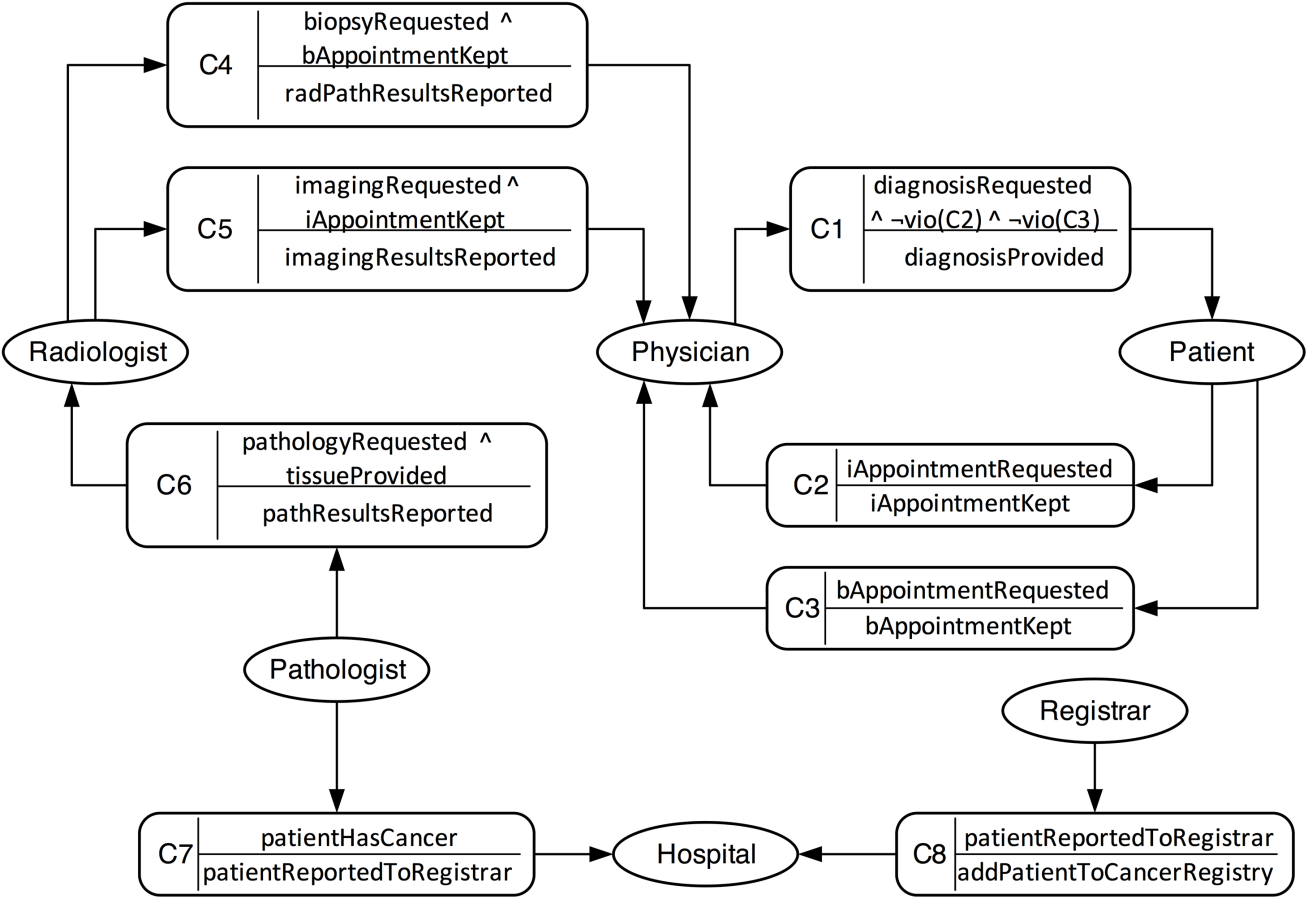
A Comma business model for the ASPE scenario.

We now describe the Comma solution for the breast cancer scenario. For brevity, we omit the outputs of the intermediate Comma steps. Fig 3 shows the resulting business model, comprising eight commitments. The figure employs Telang and Singh’s [5] notation. An oval represents a role (with a name). A rounded rectangle represents a commitment with a name on the left, its antecedent on the top right and its consequent on the bottom right. Directed edges connect a commitment’s debtor to the commitment and the commitment to its creditor.

*C*_1_ physician commits to patient to produce a diagnosis upon patient’s request, and provided patient keeps her imaging and biopsy appointments.
*C*_2_ patient commits to physician that if physician requests an imaging appointment with radiologist for her, she will keep the appointment.
*C*_3_ patient commits to physician that if physician requests a biopsy appointment with radiologist for her, she will keep the appointment.
*C*_4_ radiologist commits to physician to producing an integrated radiology and pathology report, provided physician requests a biopsy, and patient keeps her biopsy appointment.
*C*_5_ radiologist commits to physician to producing an imaging report, provided physician requests imaging and patient keeps her imaging appointment.
*C*_6_ pathologist commits to radiologist to producing a pathology report, provided radiologist requests it and provides a tissue sample.
*C*_7_ pathologist commits to hospital to report a cancer-diagnosed patient to registrar.
*C*_8_ registrar commits to hospital to adding patient to the cancer registry provided pathologist reports patient has cancer.

Note that above we introduce a role, hospital, and assume that pathologist and registrar commit to hospital for reporting and adding patient to the cancer registry. If a legal requirement mandates adding a patient diagnosed with cancer to the registry, pathologist and registrar may commit to another role government instead of to hospital.

**Fig 4:**
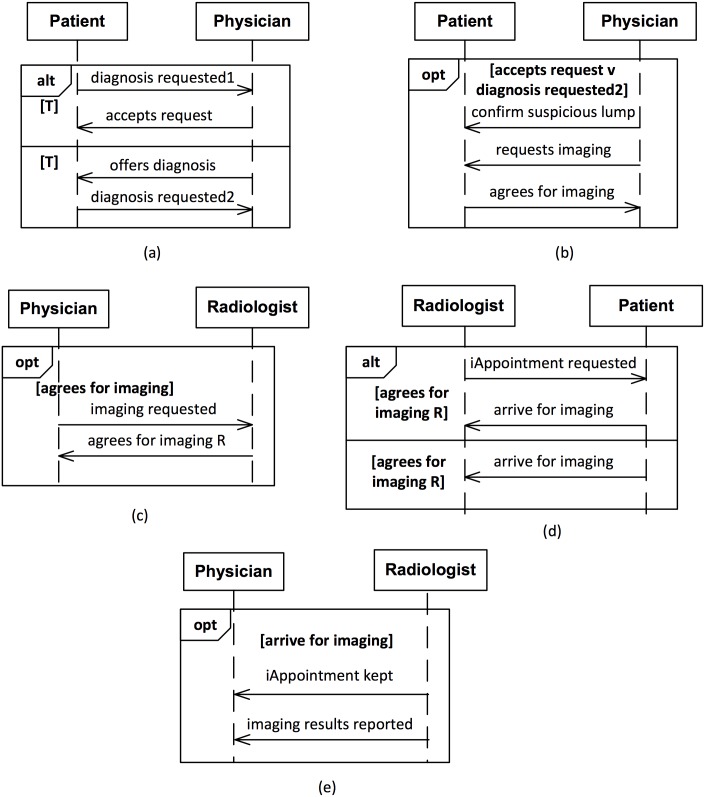
Representative SDs from the Comma-produced operational solution for the ASPE scenario. (a) patient requests physician to perform diagnosis. (b) physician requests patient to go for imaging. (c) physician requests radiologist to perform imaging. (d) physician arrives for imaging. (e) radiologist reports imaging results to physician.

Fig 4 shows a subset of the SDs for the above Comma model. (The S1 Appendix describes the remaining SDs for the ASPE scenario.) Notice that the SDs are modular, indicating improved flexibility and comprehensibility. In Fig 4(a), physician creates commitment C_1_ toward patient by either offering to provide diagnosis or agreeing to perform one. In Fig 4(b), physician observes a suspicious lump and requests patient to obtain imaging, which patient agrees to do. By agreeing, patient creates commitment C_2_ toward physician. In Fig 4(c), radiologist requests patient to arrive for imaging. radiologist agrees to doing so and creates C_5_. patient arrives at radiologist’s office for imaging, thus satisfying C_2_ by keeping her imaging appointment. Finally, radiologist sends the imaging report to physician, and satisfies C_5_.

The above exercise demonstrates the potential benefits of Comma over Traditional-HL7. The Traditional-HL7 approach typically leads to large and difficult-to-comprehend sequence diagrams, as in Fig 2. Further, the SDs produced using Traditional-HL7 typically encode only a few enactments, and disallow many other enactments that may be acceptable at a business level. That is, the Traditional-HL7 SDs restrict participant flexibility. In contrast, Comma produces modular and smaller SDs that are easier to comprehend. Comma SDs promote participants’ flexibility by modeling enactments that are acceptable at a business level. For example, in the Comma SD from Fig 4(a), either patient can request physician to perform diagnosis, or alternatively physician may offer patient to perform diagnosis. In contrast, in the Traditional-HL7 SD from Fig 2, physician cannot offer to perform diagnosis. This is because Traditional-HL7 models express operational details directly whereas Comma models express the underlying business relationships and from those relationships derive the operational details. Besides superior SDs, Comma produces a formal commitment-based business model that serves as a basis for (automatically) verifying operational models.

### Part Two: Empirical Evaluation

The foregoing assessment identifies the potential benefits of Comma over Traditional-HL7. It would be valuable to determine whether such benefits would obtain when both approaches are applied by independent modelers.

We compared the effectiveness of Comma and Traditional-HL7 approaches via an empirical study of 47 subjects. These subjects were graduate students in computer science, several with prior industry experience as developers: eleven had more than five years; 27 had one to four years; and nine had none. The study used a *between-subject* experimental design [17]. For each exercise, the study divided the subjects into two groups, who applied different methodologies to model the same scenario. The exercises were a part of class assignments, and the subjects had two weeks time to complete each of the exercise. The subjects were thus amply motivated to produce their best work. We provided a description with about the same level of detail of each methodology to the subjects.

We performed the study in two phases. In the first phase, we partitioned the subjects into two groups, Group *A* and Group *B*. The subjects in Group *A* built SDs for the ASPE scenario using HL7 messages and those in Group *B* did the same using Comma. Each subject worked individually.

In the second phase, we partitioned the subjects into two new groups, Group *C* and Group *D*, each with half of its members drawn from Group *A* and half from Group *B*. We developed Traditional-HL7 and Comma solutions for the first phase. We provided Group *C* subjects our Traditional-HL7 solution, and Group *D* subjects our Comma solution. Each subject worked individually to modify the provided model to accommodate the specified changes to the scenario. (The S1 Appendix describes the changes to the scenario we provided.)

**Ethics:** We followed the standard Institutional Review Board (IRB) approval process established by the NC State University. The NC State University IRB has approved our study. The participants provided written informed consent to participate in this study. Note that our study was not clinical in nature. It involved creating software models using two approaches.

Our study design addresses the following internal threats to validity [17].

- It **balances the subjects’ skill-sets** by creating groups and subgroups with equal mean expertise (calculated based on their educational background, business process modeling, and software development experience, as obtained via a qualifying survey). Other than balancing the groups on expertise, the subjects were randomly assigned to the groups.
- It mitigates subjects’ **learning effect** by balancing Groups *C* and *D* with respect to experience in Phase 1 with Traditional-HL7 and Comma. Moreover, it requires each subject to work individually without communicating with others.
- It eliminates **the instrumentation difference** by having subjects use the same tools; developing SDs using IBM RSA v8.0 and Comma models using an Eclipse-based tool [18] (available for download at: http://research.csc.ncsu.edu/mas/code/Protos).
- It prevented the subjects to **learn from each other** by requiring them to sign an honor code to not collaborate. Further, we confirmed that the subjects actually did not collaborate–the models produced by different subjects were different based upon features such as SD count, message count, and message names.
- It reduced the **authors’ bias** in the subjective quality assessment of the models through the use of a grading rubric. The rubric additionally provided structure to the subjective assessment.

Next, we discuss the external threats to the validity of our results.

- Can the results of this study be safely generalized for scenarios other than the one used by the study?
  The study employed the *breast cancer diagnosis* scenario that is representative of processes in healthcare. Further, note that this scenario was produced and coded by a committee of the US Department of Health and Human Services, independently of Comma. A strong point in favor of this scenario is that it reflects the consensus view of several leading researchers.
  We have conducted similar studies employing scenarios from the commerce and manufacturing domains [7, 10], where we obtained similar results. We conjecture that because Comma provides high-level abstractions, it will yield greater benefits when applied to more complex scenarios than the breast cancer scenario employed in the present study. Evaluating Comma on more complex healthcare scenarios would be an interesting topic for further investigation.
- Can the results of this study apply to healthcare IT professionals, not just computer science graduate students?
  Although our study employed graduate students as the subjects, several of them had several years of industry experience (out of our 47 subjects, 11 had more than five years and 27 had one to four years of experience; only nine had none). Indeed, computer science graduate students mostly take up industry jobs upon graduation, many in the healthcare sector.
  We have attempted to bridge the gap between theory and practice by developing a tool [18], which builds on the popular Eclipse platform and is compatible with IBM Rational Software Architect, one of the major business modeling tools used in industry. Our tool supports developing Comma models and UML 2.0 Sequence Diagrams (SDs), and performs automated verification of the SDs with respect to the Comma model. Therefore, we expect that our methodology would fit into how healthcare professionals work today, should they decide to adopt it.

We employ the following measures (dependent variables) for comparing the two approaches. The measures we employ for evaluation are generic and domain independent. Our motivation is that healthcare IT should consider adopting best practices from IT in whatever domain, e.g., from the manufacturing domain. Our metrics correspond to better processes in general. Specifically, in healthcare, higher flexibility opens up additional clinical opportunities without jeopardizing correctness; higher coverage means that the process model tackles stakeholder requirements more completely than otherwise; higher precision means that the models produced include fewer superfluous components; and improved comprehensibility would mean that the model can be understood and enhanced more easily.

**Time:** (in minutes) taken to model sequence diagrams for the scenario.
**Difficulty** a subject perceives in modeling: an integer 1–5, interpreted as *extremely easy*, *easy*, *neutral*, *difficult*, and *extremely difficult*.
**Flexibility:** the number of enactments a model permits. Greater flexibility in general leads to increased choices for a participant. We employ two measures of flexibility.
**SD count:** indicates modularity and generally yields greater numbers of interleavings of messages from multiple SDs.
**Count of alternate (alt), option (opt), and parallel (par) fragments:** indicates more numerous possible enactments.
**Objective quality:** indicates the quality of a model based on the number of missing guards and the number of incorrect SDs structures.
**Number of missing guards** indicates the count of incorrect enactments.
**Number of incorrect SD structures** indicates the count of errors produced while modeling the messages.
**Subjective quality:** indicates the quality of a model based on the scenario coverage, precision, and comprehensibility.
**Scenario coverage:** *high* (covers the entire scenario), *medium*, *low*, and *very low*.
**Scenario precision:** *high* (no unnecessary messages), *medium*, *low*, and *very low*. By unnecessary message, we mean a message that is unrelated to the scenario requirements.
**Comprehensibility:** *high* (easy for a human to comprehend), *medium*, *low*, and *very low*. Each expert judged the comprehensibility of a model subjectively. Generally, the experts found models with several modular SDs more comprehensible than a model with fewer SDs.

Each subject submitted a worklog three times a week, reporting the time the subject spent and the difficulty the subject perceived. We computed model flexibility programmatically. Experts (the authors) judged the subjective model quality, reconciling any differences of opinion through discussion.

Next, we state our claims as a set of hypotheses.

H1 Comma yields more flexible models as compared to Traditional-HL7. We employ the sum of alt, opt, and par operator count and SD count as a measure of flexibility.
H2 Comma is more efficient than Traditional-HL7. We employ the mean modeling time as a measure of efficiency.
H3 Comma is easier to apply than Traditional-HL7. We employ the mean modeling difficulty as a measure of ease of use.
H4 Comma produces higher quality SDs than Traditional-HL7. We employ sum of missing guards and incorrect SD structures as a measure of SD quality.

## Results

This section describes the results of our empirical evaluation.

**Fig 5:**
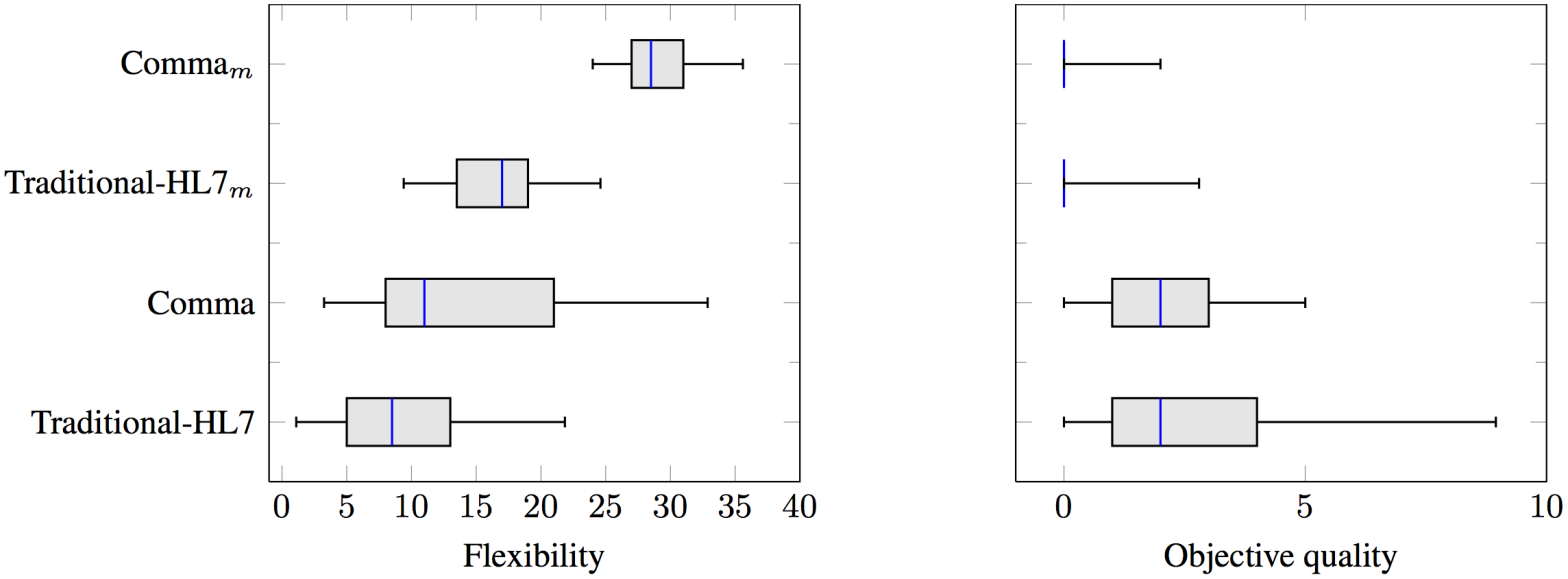
Flexibility (total number of SDs and alt, opt, par fragments) and objective quality (total number of missing guards and incorrect SDs) as assessed by experts.

Fig 5(a) shows boxplots for flexibility in terms of number of SDs and alt, opt, and par fragments. The Y-axis represents the two study phases: (1) developing sequence diagrams (Traditional-HL7 and Comma), and (2) modifying the sequence diagrams (Traditional-HL7_*M*_ and Comma_*M*_). The X-axis shows the sum of the SD and alt, opt, and par counts. Observe that the median number of SDs and alt, opt, and par fragments is higher for Comma (11) than for Traditional-HL7 (8.5). This suggests that the sequence diagrams designed using Comma are more modular and flexible than those designed using Traditional-HL7. We attribute Comma’s higher modularity and flexibility to its focus on commitments along with its reusable patterns. Further, observe that the median number of SDs and alt, opt, and par fragments for Comma_*M*_ (27) is significantly higher than the median for Traditional-HL7_*M*_ (17). We attribute Comma_*M*_’s higher values partially to the exemplar Comma solution being more modular than the Traditional-HL7 exemplar solution provided.

Fig 5(b) shows the objective quality of the SDs as assessed by domain experts. In Fig 5(b), missing guards and incorrect SDs have the same median (2) in Traditional-HL7 and Comma. This may be the result of subjects using the Comma patterns without correctly adapting them to the given scenario. For example, several subjects failed to edit the guard when applying opt pattern fragments to the correct scenario-specific value, leaving it as the default value of *true*. Additionally, observe that the median number is zero for both Traditional-HL7_*M*_ and Comma_*M*_, which we attribute to the quality of the solutions we provided in the second phase.

**Fig 6:**
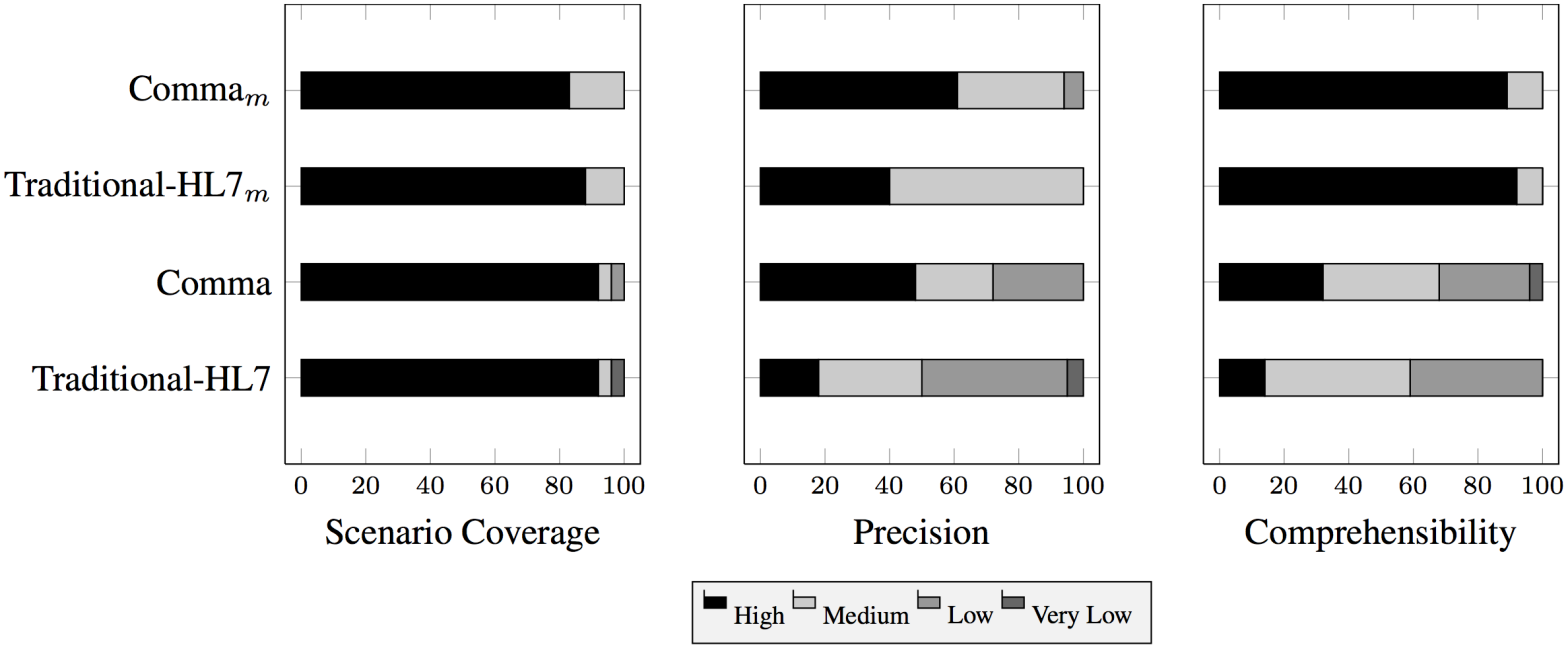
Distributions of quality, as judged by experts.

Fig 6 shows the subjective quality of the SDs judged independently by two experts. (The experts reconciled any differences through discussions.) Fig 6(a) shows the scenario coverage for the SDs. Observe that the scenario coverage is high for both Traditional-HL7 (92%) and Comma (92%), and for the modification task, the scenario coverage for Comma_*M*_ (82%) is slightly lower than Traditional-HL7_*M*_ (88%). The higher scenario coverage in both the approaches may be due to the scenario being small. Fig 6(b) shows the precision for the SDs. Observe that the Comma and Comma_*M*_ precision (40% and 61%, respectively) is higher than that of Traditional-HL7 and Traditional-HL7_*M*_ (18% and 40%, respectively). We attribute Comma’s higher precision to its systematic nature and the fact that it focuses attention on the relevant commitments. Fig 6(c) shows the comprehensibility of the models. The comprehensibility for Comma (32%) is higher than Traditional-HL7 (14%), which we attribute to Comma’s modular patterns. Further, observe that the comprehensibility for Traditional-HL7_*M*_ (92%) is slightly higher than that of Comma_*M*_ (89%).

**Fig 7:**
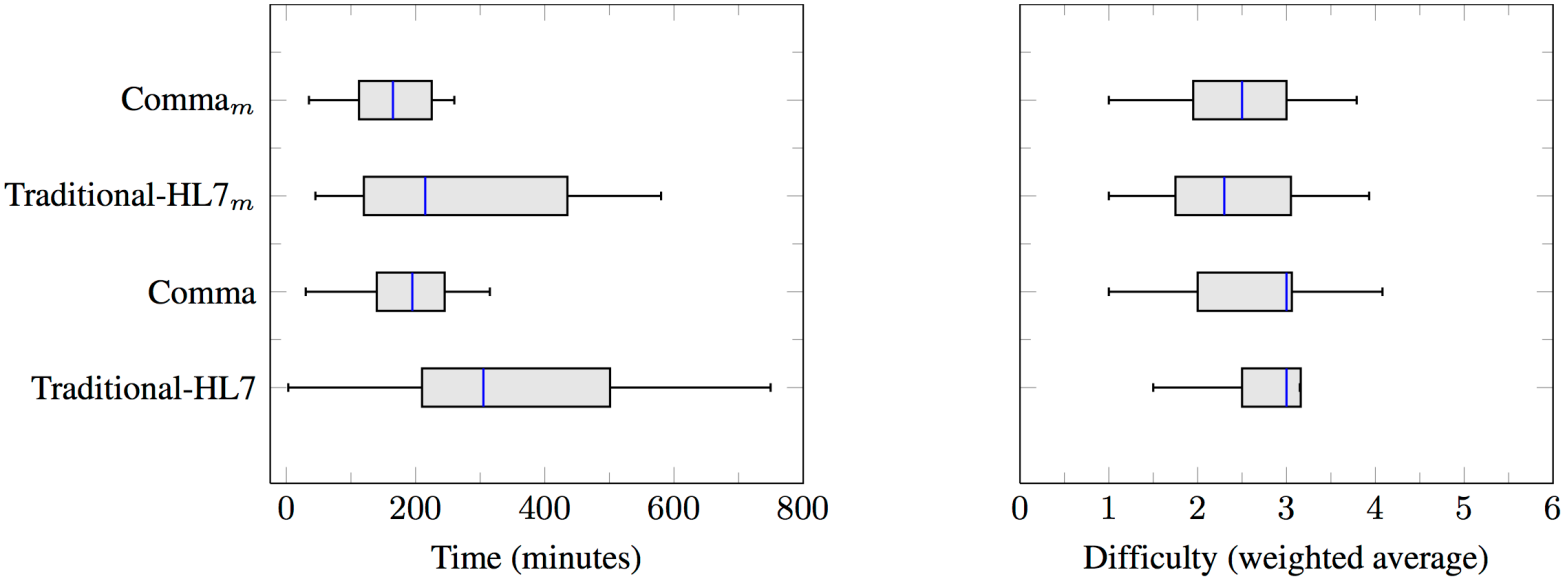
Time taken and perceived difficulty, both as reported by the subjects.

Fig 7 shows the time taken and difficulty perceived in developing the SDs using Traditional-HL7 and Comma. Fig 7(a) shows that Comma and Comma_*M*_ timings are (140 minutes and 112.5 minutes) less than Traditional-HL7 and Traditional-HL7_*M*_ timings (210 minutes and 112.5 minutes). We attribute this result to Comma’s reusable patterns and Traditional-HL7’s complexity. Fig 7(b) shows that the difficulty perceived in Comma and Comma_*M*_ (3 and 2.5) is higher than Traditional-HL7 and Traditional-HL7_*M*_ (3 and 2.3). We attribute this result to Comma’s emphasis on identifying multiple alternative enactments that are acceptable at a business level.

We now describe our hypotheses, and then present the results of hypothesis testing.

H1 Comma yields higher alt, opt, and par operator count and SD count than Traditional-HL7, which means Comma yields models that more flexible than those models that Traditional-HL7 yields.
H2 Comma takes lower mean modeling time than Traditional-HL7, which means that Comma is more efficient than Traditional-HL7.
H3 Comma subjects perceive lower mean difficulty than Traditional-HL7, which means that Comma is easier to apply than Traditional-HL7.
H4 Comma produces fewer missing guards and incorrect SD structures than Traditional-HL7, which means that Comma produces higher quality SDs with fewer incorrect enactments.

For each hypothesis, we performed the unpaired two-tailed t-test to determine whether its null hypothesis is rejected at the 10% confidence interval for the first phase of the study. As Table 4 shows, the null hypothesis is rejected for flexibility and errors, but not rejected for time and difficulty. That is, Comma models have significantly higher flexibility and lower errors (higher objective quality) compared to Traditional-HL7. However, there is no significant difference between Comma and Traditional-HL7 with respect to time and difficulty.

**Table 4:** Hypothesis testing via Student’s t-test.

Hypothesis	Traditional-HL7 (*μ*_*h*_)	Comma (*μ*_*c*_)	Null Hypothesis [*μ*_*h*_ = *μ*_*c*_] p-value	Rejected at p-value of 10%?
[H1-Flexibility]	9.59	14.4	0.0139	✓
[H2-Time]	352.14	190.00	0.6434	×
[H3-Difficulty]	2.77	2.69	0.7044	×
[H4-Errors]	2.90	2.00	0.0592	✓

## Related Literature

Zugal et al. [19] highlight that the traditional imperative approaches for process modeling lack flexibility since such approaches specify exactly how the participants should act. In contrast, declarative approaches for process modeling offer flexibility since they specify minimal constraints (only those that are necessary for a desirable business outcome) on the participants. Zugal et al. further state that declarative modeling approaches can be difficult for domain modelers to understand when they employ unintuitive abstractions. Comma addresses both challenges: it is a declarative process modeling approach that produces flexible process models. Further, Comma is based on the notion of commitments, which directly capture business process requirements in terms of interactions among the parties concerned.

Chen et al. [20] provide an approach for modeling and verifying medical processes such as for blood transfusion, which involves several parties. They define processes using the Little-JIL [21] language. Little-JIL is an imperative language for process modeling and suffers from a lack of flexibility. In contrast, Comma specifies a process model declaratively, constraining the participants minimally, and offers flexibility. A strength of the approach that Chen et al. propose is the formal verification of the process. The commitments in a Comma business model provide a basis for correctness that has been supported via formal techniques such as model checking [5].

Fox et al. [22] emphasize how to make goals explicit in a clinical process to achieve flexibility and adaptability in workflow management. Grando et al. [23] design a catalog of runtime exceptions. They use a goal-based approach for dealing with normal and exceptional workflows, expressed as keystones connected by constraints. Such approaches assume that the participant goals are known publicly. However, in an open setting, a participant’s goals are generally not public. In an open setting, only the commitments a participant makes can be treated as public. Comma business model is specified only in terms of public commitments between parties. We employ patterns to capture deviations from a normal path. For example, Singh et al.’s [24] escalation and reversion patterns handle business violations.

Grando et al. [25] support task delegation by taking into consideration the competence, responsibility, and accountability of each party involved. Responsibility and accountability map naturally to commitments. It will be interesting to incorporate an agent’s capabilities that capture its competence in our approach.

Müller et al. [26] describe the importance of interoperability in healthcare processes but focus only on data interoperability, which is insufficient. Comma provides a semantic basis for business-level interoperability, supporting design and verification. For example, when a physician accepts to diagnose a patient, the physician commits (*C*_1_) to providing a diagnosis if the patient keeps imaging and biopsy appointments upon the physician’s request. A physician and a patient are interoperable only if both of them infer this commitment (*C*_1_) when the physician accepts the patient’s request for diagnosis.

Some approaches, e.g., [27–29] model workflows and processes via temporal logic constraints on the occurrence and ordering of activities. Of relevance to healthcare, Rovani et al. [30] employ Declare, proposed by Pesic et al. [29], for analyzing medical treatment processes. These approaches exhibit the benefits of declarative approaches, namely, flexibility, modularity, and formal reasoning. A key benefit of Comma over these approaches is that not only is Comma declarative (and formal) but being based on commitments, it captures processes at a high level of abstraction. Specifically, a Comma specification does not directly constrain ordering and occurrence of activities but captures the business relationships among the concerned parties. That is, Comma addresses compliance with business relationships rather than compliance with operational details. The patterns in Comma naturally capture business scenarios such as outsourcing.

Business Process Modeling Notation (BPMN) [31] is a leading industry standard for specifying business processes. BPMN imperatively specifies a business process in terms of data and control flows leading to highly regimented processes. Unlike BPMN, Comma declaratively specifies a business process in terms of commitments among the participants. Comma offers flexibility to the participants to interact (execute a process) in numerous ways, and only requires them to discharge their commitments.

Hinge et al. [32] present an approach for detecting treatment conflicts between multiple clinical processes. They annotate a BPMN process model with semantic effect descriptions, and employ the annotations to automatically detect any conflicts. It would be interesting to study how treatment conflicts can be detected in process models specified in Comma. We conjecture that whereas a traditional process model, such as BPMN, facilitates detecting operational conflicts (based on treatment steps), Comma would be superior for detecting semantic conflicts (based on the meanings of the treatments).

The field of agent-oriented software engineering (AOSE) has garnered significant research interest and progress. AOSE approaches employ high-level abstractions such as commitments [33, 34], norms [35, 36], and goals [37, 38]. However, the adoption of AOSE research in domains such as healthcare has been hampered by a lack of empirical studies that substantiate the benefits of the proposed methods [39]. We hope that our extensive empirical study paves the adoption of agent-oriented software engineering in healthcare.

Researchers have studied *meaningful use* of standards in healthcare from the perspective of *data semantics*. As an example, Agrawal et al. [40] conduct a study to assess the data semantics of SNOMED for meaningful use in electronic health records. Our research is aimed at studying meaningful use in healthcare, but our focus is on the *process semantics*. We conjecture that there are strong connections between process modeling and data modeling that would be worthy of additional investigation.

In general, traditional approaches assume a unitary process wherein the participants do as they are told. In contrast, our approach recognizes that the participants are autonomous. Instead of specifying participants’ internal goals we specify their commitments to one another as a natural way to streamline their interactions and to support flexible enactment. In addition, traditional approaches are focused on data-level semantics, whereas Comma focuses on the process-level semantics.

## Discussion

This paper evaluates Comma business modeling methodology on modeling healthcare processes. Comma shows promising results in terms both of improving model quality and flexibility. Improved tooling would help, especially in reducing the time and difficulty involved with using Comma.

The Comma methodology is domain-independent; it can be used to model processes from various domains. Comma’s emphasis on communications and commitments potentially makes it applicable to any cross-organizational process domain. We have evaluated Comma on processes from the healthcare and manufacturing domains [10]. Comma respects the autonomy of the participants by not imposing a regimented sequence of process steps. Since Comma is technology-independent, it permits the participants to adopt any information systems technology.

This work on Comma suggests some important directions for future investigation. One theme we have begun to study is expansion of the semantics of commitments to incorporate a variant of commitments called dialectical commitments [41], which can help capture deeper semantics about certain healthcare communications, such as medical diagnoses and laboratory results. A second theme would be to mine processes from observations. Comma’s foundations in commitments can help it provide a basis for mining high-level processes, such as might be supported by communicative acts (sometimes termed “performatives”) [42, 43].

Note that our proposal is not to replace the HL7 standard or framework with Comma. Instead, we contend that an improved methodology for developing healthcare processes can be obtained by combining the HL7 standard and Comma. Comma can be employed to yield a higher quality (flexible, precise, and comprehensible) *process model*, and HL7 can be employed to develop an information model for the messages in that process model.

Although clinical practice in healthcare is based on extensive empirical studies, comparative studies of healthcare business process representations and methodologies are rare or nonexistent. This paper seeks to establish empirical studies of processes as a way of improving healthcare processes.

## Supporting Information

S1 Appendix Details on commitments, patterns, and HL7 solution. (PDF)
