# Supplementary material for: Modeling Healthcare Processes Using Commitments: An Empirical Evaluation
Source: PLoS One. 2015 Nov 5;10(11):e0141202. doi: 10.1371/journal.pone.0141202 (PMC4634947; doi:10.1371/journal.pone.0141202)
Supplement: S1 Appendix — (PDF) [file pone.0141202.s001.pdf]

Appendix–Details on commitments, patterns, and HL7 solution

Commitments in Detail

Below we describe the components of a commitment in more detail.

DEBTOR: the participant that commits to bring about the consequent condition if the antecedent condition holds. At design time, we specify DEBTOR as a role. The role is played by a participant at runtime. DEBTOR may perform the requisite action or persuade another agent to perform the action to bring about the consequent. This flexibility is a key benefit of commitments: they depend upon whether a condition is brought about; the means employed are irrelevant, unless specified. In the above example, PHYSICIAN is DEBTOR.

CREDITOR: the role (participant at runtime) for whom the DEBTOR brings about the consequent if the antecedent holds. CREDITOR may bring about the antecedent, thereby making the commitment unconditional. By making the commitment unconditional, CREDITOR sets up a situation where either the DEBTOR must bring about the consequent or the commitment would be violated (which would be undesirable in other ways). In the above example, PATIENT is CREDITOR.

ANTECEDENT: the precondition that needs to hold for the commitment to become unconditional. DEBTOR need not bring about the commitment's consequent if its antecedent is not true. Conversely, we expect that a cooperative DEBTOR will try to satisfy an unconditional commitment by bringing about its consequent. In the above example, insurance-approval is the antecedent, which means that the insurance company approves the treatment.

CONSEQUENT: the condition that DEBTOR may bring about to satisfy the commitment. Typically, DEBTOR brings about the consequent of a commitment after the antecedent holds. But this is not necessary; DEBTOR may bring about the consequent even when the antecedent is not holding. In the above example, treatment-provided is the consequent, which means that the treatment is provided to the patient.

Lifecycle Notation

In Fig. 1, the rounded rectangles represent the states, and the directed edges represent the transitions. The label on a rectangle is the commitment state it represents, and the label on an edge is an action or an event that causes the corresponding transition. The edge is directed from the start state to the end state of the transition.

Outsourcing SDs

Fig. 1 shows the SDs for the outsourcing pattern. A message labeled with a proposition (usually part of the antecedent or consequent of some commitment) brings about that proposition. A message labeled m_i for some i means an operation on some commitment (such as its creation), which we annotate on the side. In Fig. 1(a), the outsourcer sends m_1 to the client, which creates commitment C_1. The client sends payOut to the outsourcer upon receiving m_1, which detaches C_1 since it is C_1's antecedent. In Fig. 1(b), after receiving m_1, the outsourcer sends m_2 to the contractor, and after receiving m_2 the contractor sends m_3 to the outsourcer. Alternatively, the contractor first sends m_3 to the outsourcer, and after receiving m_3, the outsourcer sends m_2 to the contractor. m_2 creates C_3 and m_3 creates C_4. In Fig. 1(c), after m_2 and m_3 are exchanged, the outsourcer sends payCon to the contractor and the contractor sends m_4 to the outsourcer in either order. Now payCon satisfies C_3 and detaches C_4; and, m_4 creates C_2 and satisfies C_4. In Fig. 1(d), after m_4 is exchanged, the contractor sends task (message) to the client. This satisfies C_1 and C_2 since task is their consequent. As part of creating a model, a modeler substitutes the message labels m_i with domain-specific terms.

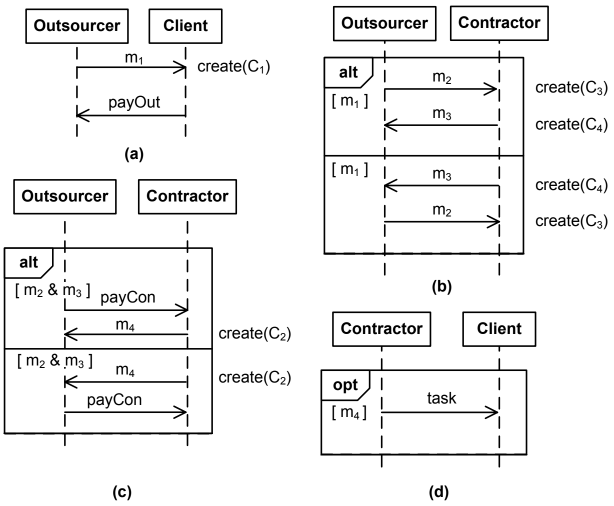

Figure 1. SDs for outsourcing.

HL7 Details

HL7 specifies two categories of message types: request response, e.g., ADT followed by an ACK (Acknowledgment), and one-sided, e.g., ORM (Order Message) and RXO (Pharmacy/Treatment Order). A trigger event is initiated when there is a need for dataflow. For example, A01 (Admit) triggers a patient's admission or visit notification. The event can cause one or more messages to be exchanged. The event can trigger additional associated events. For example, A02 (Transfer) may be followed by A08 (Update Patient Information).

HL7 lacks an extensive formal software methodology geared toward modeling processes. Table 2 shows the steps we adopted. Step 1 identifies the applicable messages from an HL7 catalog based on message "intent" as described in the catalog. Step 2 creates sequence diagrams that show the role (participant) names as lifelines and the messages they exchange.

HL7 is a consortium of over 500 major corporations that develops comprehensive standards for information exchange in healthcare including major areas such as clinical practice, and delivery of health services. The HL7 Version 2 Messaging Standard specifies messages and trigger events to support communication. Each message has a type, which describes its purpose. For example, the ADT (Admit Discharge Transfer) type includes several messages dealing with patient admission, discharge, and transfer. HL7 specifies two categories of message types: request response, e.g., ADT followed by an ACK (Acknowledgment), and one-sided, e.g., ORM (Order Message) and RXO (Pharmacy/Treatment Order). A trigger event is initiated when there is a need for dataflow. For example, A01 (Admit) triggers a patient's admission or visit notification. The event can cause one or more messages to be exchanged. The event can trigger additional associated events. For example, A02 (Transfer) may be followed by A08 (Update Patient Information).

HL7 Version 3 addresses shortcomings of the HL7 Version 2 such as a lack of information consistency. HL7 Reference Information Model (RIM) [3] is central to the HL7 Version 3. RIM addresses the lack of information consistency in HL7 Version 2, and promotes adoption of new technologies. Effectively, RIM serves as a foundation for various HL7 message standards. For developing message information schemas, HL7 Version 3 provides a methodology named HL7 Development Framework (HDF) [6], which consists of four phases. First, the modeler creates a *use case model* that captures the business requirements, and identifies a set of necessary messages. Second, the modeler creates a *domain information model* that captures the data that the messages will carry. Third, the modeler creates a *process (interaction) model* to capture the sequence of messages that the participants exchange. Fourth, the modeler creates a *message information model* that contains data relevant to a message or group of messages. The HL7 working groups employ HDF and RIM to develop messages for various domains such as Care

Provision, Cardiology, and Financial Management.

HL7 Solution Details

Observe that Fig. 2 abstracts away from system names, and shows the lifelines with the role (participant) names. In a specific realization of the sequence diagram, systems implemented by the participants would exchange the messages. For example, PHYSICIAN may implement an application employing the patient problem and acknowledge message schemas. Upon checking into the office, PHYSICIAN would request PATIENT to provide the problem details, which PHYSICIAN (or nurse) would manually enter into the application. As another example, RADIOLOGIST and PHYSICIAN may implement HL7-based systems that exchange the order imaging and imaging report messages.

Missing Guards Example

Fig. 2 shows a few examples of SDs with missing guards. In Fig. 2(a), the SD is missing a guard of collecting the PATIENT's tissue sample. Without this guard, RADIOLOGIST can incorrectly request PATHOLOGIST to test the tissue before PATIENT's tissue sample is collected. In Fig. 2(b), the guard in the second fragment of the ALT block is missing. Without this guard, instead of executing the first fragment, PHYSICIAN may execute the second fragment of the ALT block even when the tumor is benign.

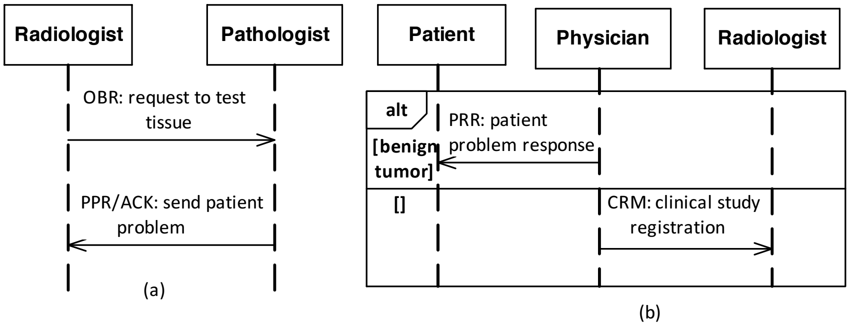

Figure 2. A few examples of SDs with missing guards.

Incorrect SD Structure Example

Fig. 3 shows a few examples of SDs with incorrect structures. In Fig. 3(a), PATIENT has no way to know if RADIOLOGIST has sent the order acknowledgment message to PHYSICIAN. Thus, PATIENT cannot request RADIOLOGIST to perform biopsy after RADIOLOGIST sends the order acknowledgment message. In Fig. 3(b), PHYSICIAN has no way to know when PATIENT sends the discharge notification acknowledgment message to RADIOLOGIST. Thus, PHYSICIAN cannot send the results to PATIENT after PATIENT sends the discharge notification acknowledgment to RADIOLOGIST.

Subjective Quality Rubric

We provided the following rubric to the experts to judge scenario coverage.

Number of scenario requirements missed	Scenario Coverage
None	High
1-2	Medium
3-5	Low
>5	Very low

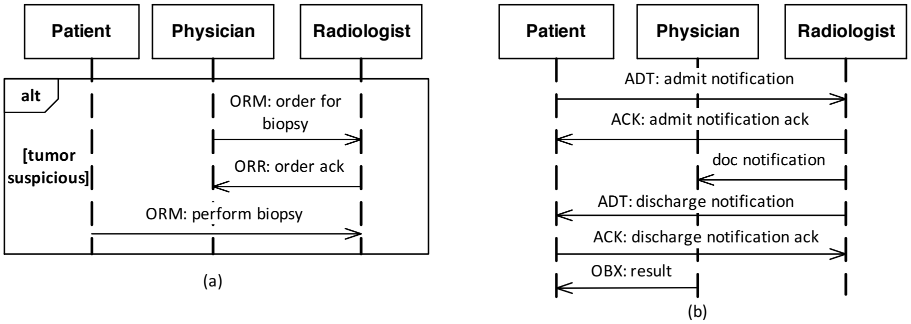

Figure 3. A few examples of SDs with incorrect structures.

We provided the following rubric to the experts to judge scenario precision.

Number of unnecessary messages	Scenario precision
None	High
1-5	Medium
6-10	Low
$>$10	Very low

Sequence Diagrams for ASPE Scenario

The Methods section describes a few SDs for the ASPE scenario. We describe the remaining SDs here.

Fig. 4 describes the biopsy scenario. In Fig. 4(a) when PHYSICIAN recommends PATIENT for biopsy, PATIENT agrees for the biopsy thereby creating C_3. Then, PHYSICIAN requests RADIOLOGIST for conducting biopsy as shown in Fig. 4(b). RADIOLOGIST agrees to it and creates C_4. In Fig. 4(c), RADIOLOGIST requests PATIENT to arrive for biopsy and PATIENT arrives. Finally, in Fig. 4(d), RADIOLOGIST notifies PATIENT's arrival to PHYSICIAN discharging C_3.

Fig. 5 describes how PATHOLOGIST and RADIOLOGIST process PATIENT's diagnosis report. In Fig. 5(a)(a), RADIOLOGIST requests PATHOLOGIST for a lab examination with PATIENT's tissue sample to which PATHOLOGIST agrees. As a result, C_6 is created by PATHOLOGIST toward RADIOLOGIST. When PATHOLOGIST provides the lab examination report to RADIOLOGIST then C_6 is discharged. Now, RADIOLOGIST takes the report from PATHOLOGIST and provides a combined Pathology-Radiology report to PHYSICIAN, declaring this to be the final report. Doing so results in discharging C_4. PHYSICIAN forwards the report to PATIENT, thereby discharging C_1.

Fig. 6 describes PATIENT's registration. If the diagnosis report provided by PHYSICIAN to PATIENT suggests PATIENT has breast cancer, then C_7 is created. When PATHOLOGIST reports PATIENT's details to REGISTRAR, then C_8 is created, as shown in Fig. 6(a). PATHOLOGIST also reports the registration to Hospital and discharges C_7. Finally, on the request of PATHOLOGIST, REGISTRAR registers PATIENT to the cancer registry and notifies Hospital, thereby discharging C_8.

Changes in the ASPE Scenario

Below are the changes to the ASPE scenario that we provided in the second phase of the study.

- Instead of ordering a pathology report directly from a pathologist, RADIOLOGIST orders the pathology report from a clerk who serves as a broker between radiologists and pathologists. The clerk finds an appropriate pathologist and orders the pathology report from the identified pathologist.

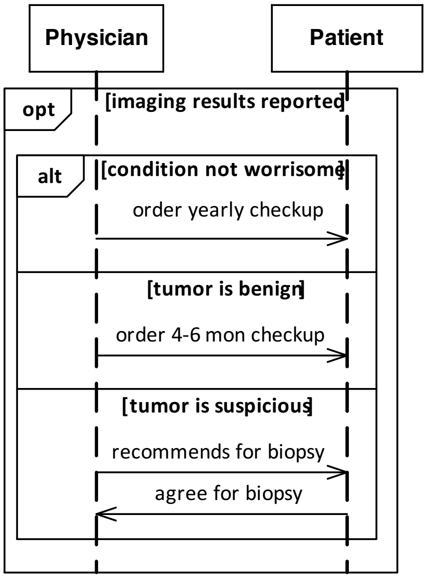

(a) PHYSICIAN recommends PATIENT for biopsy.

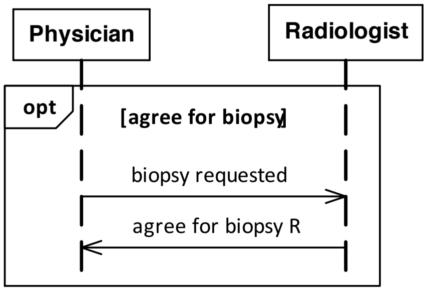

(b) PHYSICIAN requests RADIOLOGIST for biopsy.

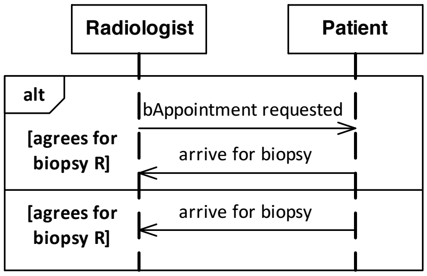

(c) PATIENT arrives for biopsy.

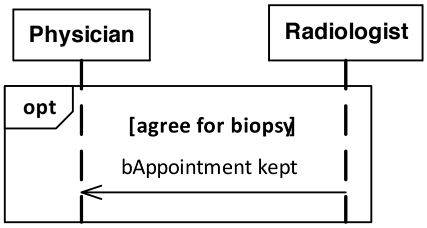

(d) RADIOLOGIST notifies PHYSICIAN of PATIENT's arrival for biopsy.

Figure 4. Biopsy scenario.

- If PATIENT is diagnosed with cancer, PHYSICIAN assigns a health coach for her. PATIENT meets the health coach twice a month for counseling and evaluation of self-management goals.

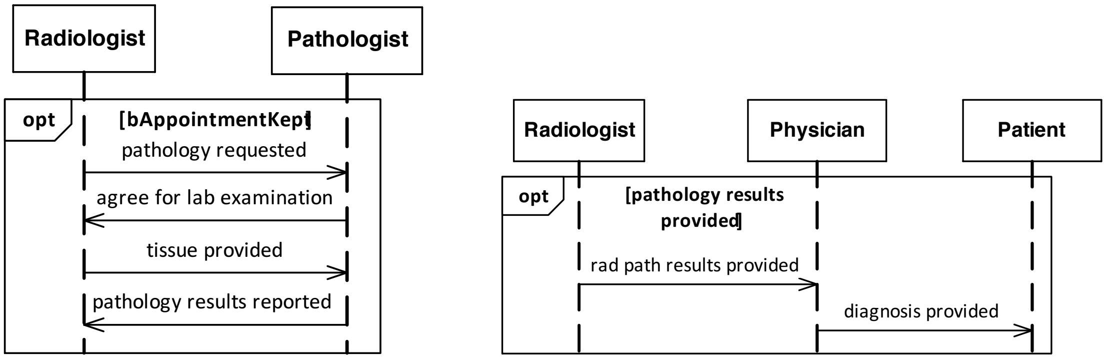

(a) RADIOLOGIST provides tissue samples to PATHOLOGIST.

(b) PHYSICIAN conveys diagnosis report from RADIOLOGIST to PATIENT.

Figure 5. Diagnosis reports.

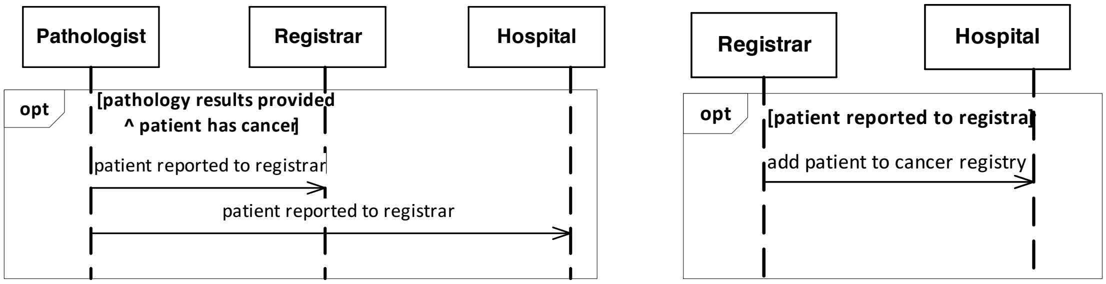

(a) PATHOLOGIST reports PATIENT's details to REGISTRAR and Hospital.

(b) REGISTRAR registers PATIENT to cancer registry.

Figure 6. PATIENT's registration.
